# Reversibility of Frailty after Lung Transplantation

**DOI:** 10.1155/2020/3239495

**Published:** 2020-08-07

**Authors:** Elyn Montgomery, Peter S. Macdonald, Phillip J. Newton, Sungwon Chang, Kay Wilhelm, Sunita R. Jha, Monique Malouf

**Affiliations:** ^1^Faculty of Health, University of Technology Sydney, Sydney, NSW, Australia; ^2^Heart & Lung Transplant Program, St Vincent's Hospital, Sydney, NSW, Australia; ^3^Western Sydney University, School of Nursing and Midwifery, Sydney, NSW, Australia

## Abstract

**Background:**

Frailty contributes to increased morbidity and mortality in patients referred for and undergoing lung transplantation (LTX). The study aim was to determine if frailty is reversible after LTX in those classified as frail at LTX evaluation.

**Methods:**

Consecutive LTX recipients were included. All patients underwent modified physical frailty assessment during LTX evaluation. For patients assessed as frail, frailty was reassessed on completion of the post-LTX rehabilitation program. Frailty was defined by the presence of ≥ 3 domains of the modified Fried Frailty Phenotype (mFFP).

**Results:**

We performed 166 lung transplants (frail patients, *n* = 27, 16%). Eighteen of the 27 frail patients have undergone frailty reassessment. Eight frail patients died, and one interstate recipient did not return for reassessment. In the 18 (66%) patients reassessed, there was an overall reduction in their frailty score post-LTX ((3.4 ± 0.6 to 1.0 ± 0.7), *p* < 0.001) with 17/18 (94%) no longer classified as frail. Improvements were seen in the following frailty domains: exhaustion, mobility, appetite, and activity. Handgrip strength did not improve posttransplant.

**Conclusions:**

Physical frailty was largely reversible following LTX, underscoring the importance of considering frailty a dynamic, not a fixed, entity. Further work is needed to identify those patients whose frailty is modifiable and establish specific interventions to improve frailty.

## 1. Background

Lung transplantation is a complex treatment and is associated with a significant risk of adverse health outcomes including infection, rejection, and death [1]. The International Society of Heart and Lung Transplantation recognizes the importance of appropriate candidate selection as a determinant of lung transplant outcomes [2]. It is essential that physiological contributors, including frailty, be considered in the risk stratification of patients undergoing transplant evaluation [3].

Frailty is a clinical syndrome characterized by decreased reserve across multiple physiologic systems. Frailty syndrome is prevalent in community-dwelling elders and across various chronic disease populations [4–6]. Frailty is predictive of short- and long-term morbidity and mortality in numerous medical and surgical populations [7,8]. Frailty assessment provides an effective method of risk stratification in several patient populations, including those with advanced lung disease referred for transplantation [6, 7]. Frailty has been highlighted as an important consideration in the assessment of lung transplant candidates across all ages [3]. Our previous reports in patients referred for heart or lung transplantation demonstrate that frailty is associated with increased mortality on the waitlist and following transplantation [4, 7, 9].

Pretransplant frailty assessment improves risk stratification and helps refine candidate selection [6]. However, currently, there is no international standard frailty measure. A number of frailty measures exist and are used for population screening or clinical assessment [10]. The two most common frailty measures are Fried's Phenotype and Rockwood's frailty index [5, 11]. Questions remain about the optimal frailty measure in lung transplantation and which frailty domains are most amenable to intervention pre- and post-lung transplantation. Physical frailty most often assessed using Fried's Frailty Phenotype is prevalent in LTX candidates and incorporates components, such as exhaustion and slow gait speed, that are likely to improve with LTX [3]. The frailty index is associated with lower posttransplant survival [12]. However, the frailty index is less likely to be disease-specific and improve following LTX. In fact, the frailty index has the potential to worsen with the addition of posttransplant comorbidities, such as diabetes [3]. As frailty measures in lung transplantation develop, it is important to ensure that the frailty domains being measured are associated with the biological processes underlying advanced lung disease.

It has been reported that the implementation of appropriate interventions may improve frailty and mitigate further decline [13]. Prehabilitation includes exercise, nutritional, and psychological components and is intended to enhance functional capacity in preparation for future physiologic stressors, such as surgery [14–16]. Pilot studies have demonstrated that prehabilitation programs are capable of improving frailty in transplant candidates and may potentially improve posttransplant outcomes [15, 16]. A recent consensus statement on frailty in transplantation recognized exercise and nutritional interventions as areas for future study in the management of frailty in lung transplantation [3]. Despite the high risk of adverse outcomes among frail patients, there may be the potential for frailty to improve prior to and following LTX [17].

Given the prevalence of frailty among those with advanced lung disease referred for transplantation, our study aimed at determining to what degree frailty is reversible following LTX in those classified as frail at the time of LTX evaluation and which specific domains of physical frailty are reversible.

## 2. Materials and Methods

### 2.1. Study Population

Between March 2013 and October 2017, 166 consecutive LTX recipients at our center were included in the study. All patients underwent frailty, cognition, and depression assessment as part of the routine evaluation for LTX. All patients undergoing evaluation for LTX are required to enroll in their local pulmonary rehabilitation program and continue pulmonary rehabilitation while awaiting LTX. Pulmonary rehabilitation was based on the Australian and New Zealand Pulmonary Rehabilitation Guidelines [18]. Patients underwent an initial assessment followed by individualized exercise training focused on increasing physical function and education to help patients manage their condition.

Following LTX, all recipients were enrolled in a 12-week outpatient rehabilitation program at our center once they have been discharged following LTX. Participants underwent an initial assessment (six-minute walk distance and health-related quality of life assessment) with programs tailored to the individual consisting of sessions of upper and lower extremity exercise, education, and psychosocial support. Based on our experience in heart transplant recipients, we anticipated that frail patients would face a higher mortality after transplant, and in those that survived, frailty would largely be reversed. As such, during the study period, 18 patients assessed as frail pretransplant underwent reassessment of frailty, cognition, and depression on completion of the rehabilitation program following LTX.

St Vincent's Hospital Human Research Ethics Committee approved the study (LNR/13/SVH/21). Informed consent was obtained from all patients for their data to be entered into the study database for analysis.

### 2.2. Frailty Assessment

Physical frailty was assessed using a modified version of Fried's Frailty Phenotype to categorize patients as frail or nonfrail. Details of the modified assessment tool have previously been published [19] and are outlined in Table 1. Patients were assessed as frail if ≥ 3 domains of the modified Fried Frailty Phenotype (mFFP) were present.

### 2.3. Cognition and Depression Assessment

The Montreal Cognitive Assessment (MOCA) questionnaire was used to assess cognitive function [20], with a score less than 26/30 classified as cognitive impairment.

The Depression in the Medically Ill (DMI-10) [21] questionnaire was used to assess depression. A score of  ≥ 9/30 was classified as a likely case of clinical depression.

### 2.4. Hand Grip Strength

Hand grip strength (HGS) was assessed using the Jamar Hand Dynamometer. Grip strength was considered weak if the average of 3 consecutive attempts on the left and right hand was less than 2 standard deviations below the sex- and age-adjusted normal values. The higher average of the right-hand and left-hand averages was recorded for baseline and follow-up HGS.

### 2.5. Assessment of Disease Severity

Markers of lung disease severity were obtained as part of the routine pretransplantation evaluation. These prognostic markers included PaO2 levels, FEV1, FEV1 % predicted, DLCO, DLCO % predicted, FVC, and FVC % predicted. Biochemical parameters were obtained including blood haemoglobin level, serum creatinine, serum albumin, serum bilirubin, presence of anemia (male: haemoglobin <130 g/L; female: haemoglobin <115 g/L), and presence of hypoalbuminemia (serum albumin <35 g/L), and estimated glomerular filtration rate (eGFR) was calculated using the modification of diet in renal disease formula. Body mass index (BMI) was calculated as weight/height [2] (kg/m^2^).

### 2.6. Outcome Measures Morbidity and Mortality

Post-LTX intubation time, intensive care unit (ICU) length of stay (LOS), hospital LOS, and 12-month post-LTX survival were recorded for all patients.

### 2.7. Assessment of Reversibility of Frailty

For patients assessed as frail pre-LTX, follow-up frailty assessment, cognition assessment, and depression screening were performed following completion of the post-LTX rehabilitation program.

### 2.8. Statistical Analysis

Descriptive statistics were calculated for all variables. The number of patients within the nonfrail and frail categories was determined for the study population. Baseline characteristics are presented as mean ± standard deviation or median and interquartile range for continuous variables and frequency (percent) for categorical variables. The association between frailty category and age, sex, transplant type, diagnostic category, cognition, depression, markers of lung disease severity, and biochemical parameters was made using independent-sample *t*-tests or Mann–Whitney *U* tests for continuous variables and chi-square tests or where appropriate Fisher's exact test for categorical variables.

To compare outcomes, survival time was defined as the time from the date of LTX and the date of death or date of censoring (12 months post-LTX). Kaplan–Meier cumulative survival curves were generated for each frailty category, and the log-rank test was used to compare 12-month survival rates between the frail and nonfrail groups. Post-LTX intubation time was defined as “the time from intubation to the time of extubation following LTX,” ICU LOS was defined as “the time from the date of transplant admission to ICU to the date of discharge from ICU,” and hospital LOS was defined as “the time from the date of transplant admission to the date of discharge from hospital following LTX.” The association between frailty category and post-LTX intubation time, ICU LOS, and hospital LOS was made using Mann–Whitney *U* tests.

To assess changes in physical frailty, individual frailty domains including exhaustion, mobility, appetite, activity, and HGS were compared at baseline and at follow-up. In addition, depression and cognition were compared at baseline and at follow-up. Related sample Wilcoxon signed-rank test was used for continuous data and McNemar's test for categorical data. A *p* value of <0.05 was considered statistically significant. All data analyses were conducted using IBM SPSS, version 25 (IBM Corp., Armonk, NY).

## 3. Results

### 3.1. Frailty Prevalence

During the study period, 166 patients (90M: 76F; age 54 (IQR 21) years, range 16–70) underwent lung transplantation (159 bilateral LTX, 4 single LTX, and 3 heart-lung transplants). The underlying causes of lung disease were cystic fibrosis (23%), chronic obstructive pulmonary disease including alpha-1 antitrypsin deficiency (33%), interstitial lung disease (30%), pulmonary arterial hypertension (3%), congenital heart disease (2%), chronic lung allograft dysfunction (5%), and “others” (4%). The median time between pretransplant frailty assessment and transplantation was 170 (IQR 220) days for frail patients and 167 (IQR 208) for nonfrail patients. Baseline demographics are provided in Table 2. Twenty-seven patients were assessed as frail (16%). Frailty was not associated with age, gender, diagnosis, BMI, PaO2, FEV1 % predicted, and DLCO % predicted. Frailty was associated with lower serum creatinine, haemoglobin and albumin, cognitive impairment (as a categorical variable) and depression (as categorical and continuous variables).

### 3.2. Frailty and Post-LTX Outcomes

**Figure 1:**
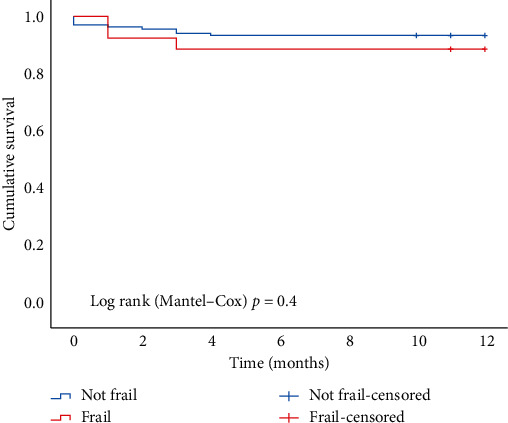
12-month post-LTX survival in frail vs. nonfrail patients.

**Figure 2:**
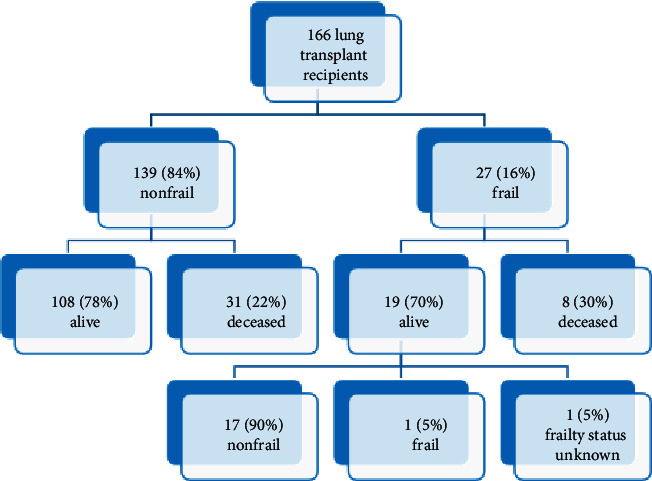
Outcome of nonfrail vs. frail patients post-LTX following reassessment of frail patients.

At 12-month post-LTX survival was 92% in the nonfrail group compared with 88% in the frail group (*p* = NS, Mantel–Cox test) (Figure 1). Post-LTX outcome for all 166 patients following reassessment of frail patients is shown in Figure 2. Post-LTX intubation time, ICU LOS, and hospital LOS outcomes by frailty status are shown in Table 3. Postoperative ICU LOS was similar for the nonfrail and frail groups.

We compared the baseline demographics for frail patients stratified by post-LTX mortality status and showed no difference between frail patients that survived versus those that died post-LTX. However, it is likely that our numbers are too small to show any significance.

### 3.3. Reversibility of Frail Pre-LTX

Of the 27 patients assessed as frail pretransplantation, 8 died following LTX and before reassessment, one interstate recipient failed to return for reassessment, and 18 underwent post-LTX frailty reassessment. Follow-up reassessments were conducted at the median time of 357 (IQR 841) days post-LTX.

Amongst the patients assessed as frail pre-LTX, there was no significant difference in the frailty scores of those who did and did not survive (alive 3.37 ± 0.6; dead 3.63 ± 0.9, NS).

**Figure 3:**
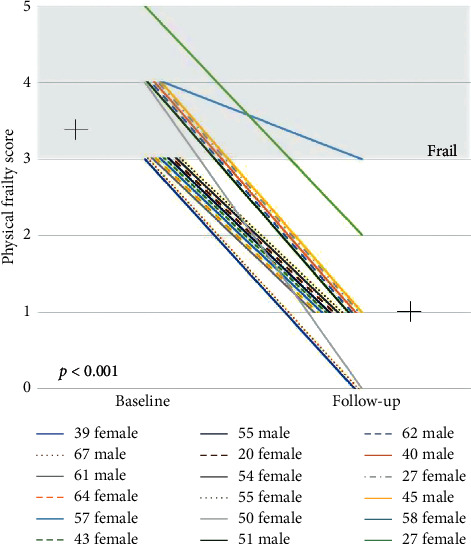
Changes to physical frailty score pre-LTX vs. post-LTX.

Amongst the surviving patients, there was a significant improvement in frailty score post-LTX (from 3.4 ± 0.6 to 1.0 ± 0.7, *p* < 0.001) (Figure 3). Of the 18 patients reassessed post-LTX, 17 (94%) were recategorized as nonfrail, while the remaining patient had an improvement in frailty score (from 4 to 3; exhaustion, poor appetite, and reduced grip strength) that did not meet the recategorization threshold.

### 3.4. Reversibility of Frailty Domains

**Figure 4:**
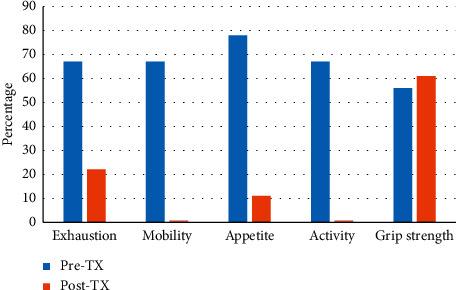
Changes to physical frailty domains pre-LTX vs. post-LTX.

Changes in the frailty domains of 18 frail patients from pre-LTX to post-LTX are shown in Figure 4.

Twelve (67%) patients met the criteria for exhaustion pre-LTX. At reassessment, there was a significant improvement (*p*=0.008) with 4 (22%) patients classified as exhausted following LTX.

Twelve (67%) patients had slow walking speed pre-LTX. There was a significant improvement (*p* < 0.001) with no (0%) patients assessed as having slow walking speed post-LTX.

Fourteen (78%) patients were classified as having poor appetite pre-LTX. At reassessment, there was significant improvement (*p* < 0.001) with 2 (11%) patients reporting poor appetite following LTX.

Twelve (67%) patients were classified as physically inactive pre-LTX. There was significant improvement (*p* < 0.001) with no (0%) patients classified as physically inactive post-LTX.

Ten (56%) patients had reduced HGS pre-LTX. At reassessment, there was no improvement (*p* = NS) in HGS. In fact, HGS worsened, with 11 (61%) patients assessed as having reduced HGS post-LTX.

### 3.5. Reversibility of Cognitive Impairment and Depression

**Figure 5:**
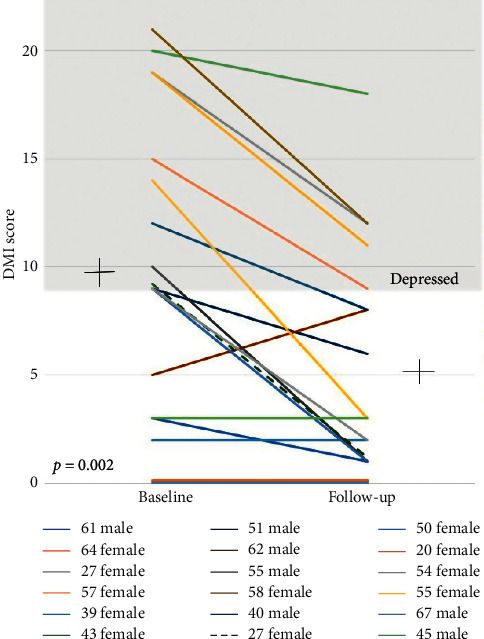
Changes in DMI-10 score pre-post LTX.

Changes in the DMI-10 of the 18 frail patients from pre-LTX to post-LTX are shown in Figure 5. Of the 13 (72%) patients classified as depressed pre-LTX, there was significant improvement in DMI-10 score (from 9.9 ± 6.9 to 5.4 ± 5.2, *p*=0.002) with 5 (28%) patients classified as depressed post-LTX.

**Figure 6:**
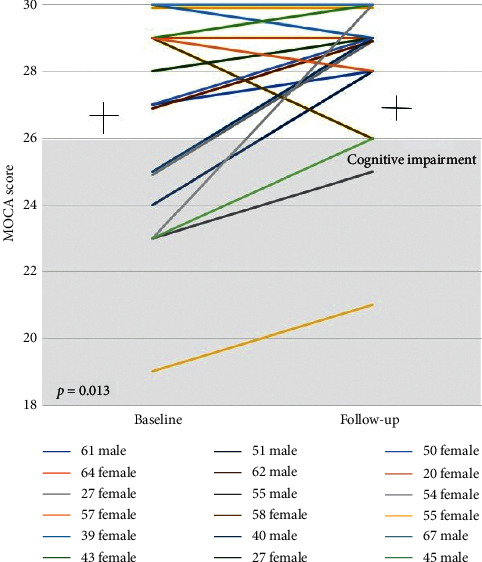
Changes in MOCA score pre-post LTX.

Changes in the MOCA of the 18 frail patients from pre-LTX to post-LTX are shown in Figure 6. Of the 7 (39%) patients classified as cognitively impaired pre-LTX, there was significant improvement in MOCA score (from 26.5 ± 3.1 to 28.1 ± 2.1, *p*=0.013) with 2 (11%) patients classified as cognitively impaired post-LTX. Although still classified as cognitively impaired, the 2 patients saw improvements in their MOCA score post-LTX (23 to 25 and 19 to 21).

## 4. Discussion

The major finding of our study is that in those who survived, physical frailty was largely reversible following lung transplantation. Of the 18 frail patients, significant improvements were seen in 17 (94%) patients (*p*=0.001). We believe this study to be one of the first to investigate the reversibility of the individual physical frailty domains following LTX.

Frailty has previously been shown to improve within the first 6 months following LTX [17]. Significant improvements were seen in the following physical frailty domains: exhaustion (*p*=0.008), mobility, appetite, and activity (all *p* < 0.001). Walk speed as assessed by six-minute walk distance and time spent in moderate-intensity physical exercise has previously been reported to significantly improve from hospital discharge to 3 months post-LTX [22]. Interestingly, exhaustion was reported to be prevalent in 56% of lung transplant recipients at routine follow-up (1–5 years post-LTX), with psychological distress the most significant predictor of exhaustion [23]. This may be due to a number of factors including post-TX mood change, cognitive problems, and unrealistic expectations of the TX process. A better understanding of the trajectory of the individual frailty domains following LTX may help clinicians determine the most appropriate prehabilitation interventions.

Reduced HGS has been suggested as a single-item surrogate measure of frailty [24] and it is therefore of interest that this was one domain where reversibility was not demonstrated post-LTX. However, these results are consistent with those we have reported in the heart transplant population [25]. In a group of 23 lung transplant recipients, Candemir and colleagues reported HGS remained lower than in healthy individuals following an 8-week outpatient pulmonary rehabilitation program [26].

Conversely, in heart failure patients undergoing ventricular assist device (VAD) placement, HGS significantly increased post-VAD implantation compared with baseline [24]. Corticosteroid-induced myopathy is well documented [27] with inverse correlations found between skeletal muscle strength and the amount of corticosteroids ingested [28]. This suggests that post-TX steroid ingestion may contribute to reduced muscle strength affecting HGS and that a comprehensive frailty tool, such as the FFP, may be more sensitive to detecting changes in frailty status over a single-item measure, such as HGS. Further research is necessary to determine the most appropriate frailty measure in lung transplantation, to determine the factors that contribute to weak HGS following the procedure and develop appropriate pre-LTX interventions.

The reversibility of frailty was first proposed by Flint and colleagues [29]. It was hypothesized that the relative proportion of disease-specific and non-disease-specific factors comprising a patient's frailty status was indicative of the potential for change, with disease-specific factors more likely to reverse following intervention.

This would suggest that, in certain patients with advanced lung disease, a number of presumably disease-specific frailty domains including walk speed, exhaustion, and physical activity would be amenable to interventions such as transplantation.

Further research is necessary in the population of frail patients with advanced lung disease to identify characteristics that are associated with an increased risk of mortality either before or after lung transplantation, thereby improving risk stratification and ensuring those selected for lung transplantation sustain a significant survival benefit [1].

Our center does not have an upper age limit for transplantation and considers frailty in conjunction with comorbidities when evaluating a patient's suitability for transplant. While we do not consider age an absolute contraindication to LTX, increasing age is generally associated with other relative contraindications, such as comorbidities, that place older LTX candidates at increased risk of morbidity and mortality following LTX [2]. While we expected that older patients would demonstrate higher rates of frailty and less reversibility of frailty than their younger counterparts, we did not find any association between age and frailty within the population of patients referred for LTX evaluation. Furthermore, in those patients that survived, we found frailty to be reversible across age groups, challenging the concern that frailty would be less reversible in older LTX recipients.

Several authors have reported recently that pre-LTX frailty is associated with increased morbidity and mortality before and after LTX [6–8,30]. Although not statistically significant, frail patients in our study demonstrated a trend towards increased hospital LOS following LTX. In light of these reports, it is clearly important to carefully consider the frailty status of all patients referred for lung transplantation [3]; however, the largely reversible nature of the frailty phenotype as demonstrated in this study and recently by others [8,17,30] supports the idea that frailty should not be considered a contraindication to transplantation.

Frailty has been associated with a decline in cognitive function, with research increasingly considering cognition in the definition of frailty [31, 32]. We have previously reported that the addition of cognitive impairment to the assessment of physical frailty increased the capacity to identify early mortality in those undergoing evaluation for heart transplant [9]. A recent report on frailty in solid organ transplantation recognized additional factors may contribute to frailty in transplant candidates and suggested the MOCA be used to assess cognition in the evaluation of frailty in lung transplant candidates [3]. Using the MOCA to assess cognitive function pre- and post-LTX, we saw significant improvements in cognitive function following transplantation (MOCA score from 26.5 ± 3.1 to 28.1 ± 2.1, *p*=0.013). In another study, the MOCA was used to assess cognitive impairment in a cohort of 47 LTX with 21 (45%) exhibiting neurocognitive impairment pretransplantation [33]. After transplantation, 27 (57%) recipients exhibited impairment in the early postoperative period and 19 (40%) participants continued to exhibit impairment at a 3-month follow-up. Comparable results were reported in a study of 49 lung transplant recipients, which demonstrated that one-third of patients experienced significant cognitive impairment from baseline to posttransplant [34]. However, a subset of older, less educated patients demonstrated significant cognitive decline from pretransplant to 6 months posttransplant [34]. In addition, among individuals with a diagnosis other than cystic fibrosis who experienced postoperative delirium, cognitive function was poorer than among nondelirious patients at 3 months following lung transplantation [33]. Among patients with cystic fibrosis, cognitive improvements were seen 3 months posttransplant and attributed to improvements in hypoxia and nonspecific factors such as fatigue [33].

Depression has been related to hippocampal atrophy and subsequent mild cognitive impairment as well as the development or worsening of the physical frailty syndrome [32]. The DMI-10 tool has been established as a valid screening tool that was designed to avoid symptoms (e.g., poor concentration and loss of appetite) common to medical illness [21]. Our study demonstrated a significant association between frailty and depression pretransplant (*p*=0.001). Significant improvements (*p*=0.001) were seen in depression post-LTX, with 8 out of 13 patients reclassified as “not depressed”. In their meta-analysis, Soysal et al. [35] concluded that there was a bidirectional relationship between depression and frailty, and one may be a risk factor for the development of the other. Cognitive impairment and depression are common amongst lung transplant candidates and may persist or worsen following LTX [33,36]. A number of frailty measures now incorporate psychosocial domains of frailty [10].

Further research is needed to provide insight into how current frailty measures may be modified to better capture frailty and predict outcomes in lung transplant candidates and recipients. Given the prevalence of cognitive impairment and depression in lung transplant candidates, a more comprehensive frailty measure may be required to provide clear indicators of which lung transplant recipients are no longer “frail” following transplant. The trajectory of frailty, cognitive function, and depression before and after lung transplantation is largely unknown [34]. Further research is needed to examine the interaction between frailty, cognitive impairment, and depression in lung transplantation and determine the most appropriate frailty measure in this population.

Our findings contribute to the evidence demonstrating that frailty associated with advanced disease is largely reversible following transplantation. A single-center study of 246 LTX recipients demonstrated that frailty measured by short physical performance battery (SPPB) and FFP improved early after transplant and subsequently remained stable [17]. Following bridge to transplantation VAD implantation, or heart transplantation, 24 out of 26 patients showed significant improvements in their frailty scores [25]. In a cohort of 349 kidney transplant recipients, frailty worsened at 1 month post-TX before significantly improving at 3 months [37].

Similarly, in a group of 214 liver transplant recipients, their frailty scores worsened at 3 months post-TX and improved modestly by 12 months [38]. This would suggest it is premature to delist a patient based purely on their frailty status alone and emphasizes the importance of considering frailty in combination with other comorbidities. Moreover, this underscores the need to identify and implement specific interventions to improve frailty.

Research has begun to focus on interventions to moderate the risks of frailty [16,39,40]. Frailty was reported to be reversible in a group of chronic obstructive pulmonary disease patients following pulmonary rehabilitation completion [40]. Studies examining exercise training before and after lung transplantation reported significant improvements in exercise capacity, muscle strength, and physical frailty score [16,41]. These findings support the theory that frailty is largely responsive to intervention [42] and provide strong evidence to further explore the optimal management of frail patients through appropriate interventions. Further research on pulmonary rehabilitation, nutritional supplementation, and psychosocial involvement may help establish appropriate interventions to address frailty before and after LTX [3].

## 5. Limitations

Our study has several limitations. Firstly, the study is from a single center with a small number of patients.

Secondly, our study aims to determine whether frailty identified pre-LTX is reversible posttransplant and does not assess those classified as nonfrail pre-LTX. As our program is fully funded by the Australian public healthcare system, we have a finite number of resources and our patients reside across a large geographical area. As such, we focused on the frail group rather than the total transplant population.

Thirdly, there was a variable duration between baseline frailty assessment and LTX, but this is inevitable given that the time of transplantation cannot be predicted.

However, all patients are reviewed every 3 months by the LTX physicians at our center and any concern regarding deterioration in an individual patient leads to reassessment of their frailty and suitability for transplantation in conjunction with other comorbidities. During the study period, none of the patients required reassessment for LTX suitability.

Fourthly, while all patients were reassessed following completion of the mandatory post-LTX rehabilitation program following discharge, there was considerable variation in the timing of this post-LTX frailty assessment. Despite this limitation, the timing of the post-LTX frailty assessment did not affect the critical findings of post-LTX reversibility.

Furthermore, there is survivor bias, as frail patients must survive to transplant and frailty reassessment. Nonetheless, it is noteworthy that the posttransplant survival of frail patients was 88% at one year and not significantly inferior to that of nonfrail lung transplant recipients. Moreover, the posttransplant survival of both frail and nonfrail patients in our program compares favourably to the posttransplant survival of lung transplant recipients reported by the Registry of the International Society for Heart and Lung Transplantation [2].

A further limitation to our study is the use of a modified frailty tool. Our unit evaluates patients for both heart and lung transplants. This tool is used in all patients referred to our unit: it is simple to administer and clinically feasible for our population. However, it has been validated in the advanced heart failure population [19] but not yet been validated in the LTX population.

## 6. Conclusions

In those assessed as frail during LTX evaluation, frailty was largely reversible following LTX in those patients who survived to reassessment. Improvements were seen in the physical frailty domains of exhaustion, walking speed, appetite, and physical activity. There was no improvement in grip strength. This underscores the importance of considering frailty as a dynamic, not a fixed, entity. In addition, there was also an improvement in depression and cognition scores in a number of patients following LTX, but a small subset showed a cognitive decline.

Patients referred for transplantation may greatly benefit from early intervention. Prehabilitation with an emphasis on intervention before surgery may impact function and optimize postoperative recovery [43]. Further work is needed to identify those patients whose frailty is modifiable and establish specific interventions to target frailty.

## Figures and Tables

**Table 1:** Criteria for the modified Fried Frailty Phenotype (mFFP) binary score. A total of 5 physical domains were assigned 1 point if present or 0 if absent with FFP scores ≥3 considered frail and FFP = 0–2 considered nonfrail.

Domain	Scoring criteria
Exhaustion	“In the last week, did you feel on at least three days, that everything you did was an effort?” and “In the last week, did you feel on at least three days, that you could not get going?” A response of “yes” to either question met the criteria for exhaustion
Weakness (i.e., low grip strength)	Grip strength was considered weak if the average of three consecutive attempts on the left and right hand fell below two standard deviations of sex- and age-adjusted normative values
Mobility, i.e., slow gait speed	Walking speed was considered slow if the average of three attempts took six seconds or more to complete 5 meters
Appetite	“Have you, in the last three months, been eating more/less than usual?” A response of “less” was classified as poor appetite
Physical activity	“How often do you engage in activities that require a low or moderate level of energy, such as gardening, cleaning the car or going for a walk?” A response of “one to three times a month or hardly ever” was classified as physical inactivity

**Table 2:** Comparison of demographics and baseline prognostic markers of a study population stratified by physical frailty status.

	Total (*n* = 166)	Nonfrail (*n* = 139)	Frail (*n* = 27)	*p* value
Age (years)	54 (21)	54 (21)	54 (22)	NS
Gender, male (%)	90 (54%)	79 (57%)	11 (41%)	NS
Diagnosis				
CF	39 (23%)	35 (25%)	4 (15%)	NS
COPD/AAT	54 (33%)	48 (35%)	6 (22%)	
ILD	50 (30%)	39 (28%)	11 (40%)	
PAH	5 (3%)	4 (3%)	1 (4%)	
CLAD	8 (5%)	4 (3%)	4 (15%)	
CHD	4 (2%)	3 (2%)	1 (4%)	
Others	6 (4%)	6 (4%)	0	
Transplant type				
Bilateral lung	159 (96%)	134 (96%)	25 (93%)	NS
Single lung	4 (2.4%)	2 (1.4%)	2 (7%)	
Heart-lung	3 (1.6%)	3 (2.6%)	0	
Serum creatinine (*μ*mol/L)	73 ± 21	75 ± 21	65 ± 19	0.02
eGFR (ml/min/m^2^)	82 ± 12	82 ± 12	84 ± 11	NS
Serum bilirubin (*μ*mol/L)	10 ± 9	10 ± 10	6 ± 4	NS
Serum albumin (g/L)	43 ± 5	44 ± 5	40 ± 6	<0.001
Hypoalbuminaemia, *n* (%)	8 (5%)	4 (3%)	4 (15%)	0.03
Haemoglobin (g/L)	141 ± 19	143 ± 18	129 ± 22	0.001
Anemia, *n* (%)	18 (11%)	10 (7%)	8 (30%)	0.003
Abnormal MOCA, *n* (%)	42 (25%)	31 (22%)	11 (41%)	0.04
Abnormal DMI, *n* (%)	48 (29%)	33 (24%)	15 (56%)	0.001
PaO2 (mmHg)	64 ± 11	64 ± 10	64 ± 15	NS
FEV1 (% predicted)	39 ± 21	39 ± 21	39 ± 19	NS
FVC (% predicted)	65 ± 20	67 ± 19	56 ± 17	0.008

**Table 3:** Comparison of clinical characteristics post-LTX of a study population stratified by physical frailty status.

	Total (*n* = 166)	Nonfrail (*n* = 139)	Frail (*n* = 27)	*p* value
	Median (IQR)			
Intubation post-LTX (hours)	24 (69)	23 (71)	31 (66)	NS
ICU LOS (days)	5 (7)	5 (6)	4 (9)	NS
Hospital LOS (days)	20 (21)	19 (19)	24 (30)	NS

Values are median (interquartile range) for nonnormally distributed continuous data.

## Data Availability

The data used to support the findings of this study are available from the corresponding author upon request.

## Abbreviations

BMI: Body mass index
DLCO: Diffusing capacity of the lung for carbon monoxide
DMI-10: Depression in the Medically Ill questionnaire
eGFR: Estimated glomerular filtration rate
FEV1: Forced expiratory volume-one second
FFP: Fried Frailty Phenotype
FVC: Forced vital capacity
HGS: Hand grip strength
ICU: Intensive care unit
IQR: Interquartile range
LOS: Length of stay
LTX: Lung transplantation
mFFP: Modified Fried Frailty Phenotype
MOCA: Montreal Cognitive Assessment
NS: Nonsignificant
PaO2: Arterial partial pressure of oxygen
POD: Postoperative days
SPPB: Short physical performance battery
TLC: Total lung capacity
VAD: Ventricular assist device.
